# Evaluation of Laparoscopy Virtual Reality Training on the Improvement of Trainees’ Surgical Skills

**DOI:** 10.3390/medicina57020130

**Published:** 2021-02-02

**Authors:** Mohamed Elessawy, Mohamed Mabrouk, Thorsten Heilmann, Marion Weigel, Mohamed Zidan, Ghada Abu-Sheasha, Andre Farrokh, Dirk Bauerschlag, Nicolai Maass, Mohamed Ibrahim, Dina Kamel

**Affiliations:** 1 Department of Gynecology and Obstetrics, Campus Kiel, University Hospitals Schleswig-Holstein, 24105 Kiel, Germany; thorsten.heilmann@uksh.de (T.H.); MarionTina.vanMackelenbergh@uksh.de (M.W.); Andre.Farrokh@uksh.de (A.F.); Dirk.Bauerschlag@uksh.de (D.B.); Nicolai.maass@uksh.de (N.M.); 2 Cambridge Endometriosis and Endoscopic Surgery Unit (CEESU), Gynaecology Department, Cambridge University Hospitals Foundation Trust, Cambridge 01223, UK; mohamed.mabrouk2@gmail.com; 3 Alexandria Endoscopy Association (ALEXEA), 21529 Alexandria, Egypt; dr.mohamedzidan1992.alexea@gmail.com (M.Z.); mibrahim@alexea.org (M.I.); d_kamel@alexea.org (D.K.); 4 Department of Biomedical Informatics and Medical Statistics, Medical Research Institute, University of Alexandria, 21519 Alexandria, Egypt; ghada.elsayed@alexu.edu.eg

**Keywords:** laparoscopy, evaluation of virtual reality, learning curve, surgical simulation

## Abstract

*Background and objectives*: The primary objective was to evaluate the benefit of training with virtual reality simulation. The secondary objective was to describe the short-term skill acquisition obtained by simulation training and to determine the factors affecting its magnitude. *Materials and Methods*: We prospectively performed a three-stage evaluation: face, constructive, and predictive to evaluate the training with a laparoscopic simulator with haptic feedback. The participants (*n* = 63) were divided according to their level of experience into three groups: 16% residents; 46% specialists and 38% were consultants. *Results*: Face evaluation demonstrates the acceptance of the design and realism of the tasks; it showed a median score of eight (IQR 3) on a Likert scale and 54% of participants (*n* = 34) gave the tissue feedback a moderate rating. Constructive evaluation demonstrates the improvement of the participants in the training session and the ability of the designed task to distinguish the experienced from the inexperienced surgeon based on the performance score, at task I (transfer of pegs) and II (laparoscopic salpingectomy). There was an improvement in both tasks with a significant increase in score and reduction in time. The study showed that those with a high score at the pre-test recorded a high score post-test, showing a significant pair-wise comparison (*Z*) and correlation (*p*) showing a significant statistical significance (*p* < 0.001). The predictive evaluation demonstrates the beneficiary effect of training four weeks afterward on the practice of surgeons addressed with five questions. It showed an improvement regarding implementation into daily routine, performance of procedure, suturing, shortening of the operative time, and complication management. *Conclusions*: Virtual reality simulation established high ratings for both realism and training capacity, including clinical relevance, critical relevance, and maintaining training enthusiasm.

## 1. Introduction

High-risk fields such as aeronautics use simulations to train, improve performance, and maintain the credentialing of crewmembers [1]. A recent meta-analysis published by Portelli showed that virtual reality training in laparoscopic surgery improves the efficiency and quality by reducing error rates and enhancing the handling of tissues, moreover, the simulation industry has improved the quality and the realism of simulations to accept the intense responsibility to train and prepare various occupations to perform their roles, achieving the desired standards required to ensure safety [2].

In surgical training, the introduction of various training modules, from the simple laparoscopy training boxes to virtual reality simulation and advanced training on cadavers, has allowed surgeons to improve their operative skills. The current literature shows that live surgery sessions on a donated cadaver are highly rated with an overall satisfaction for surgical education, although the unresolved ethical concerns related to live surgery events remain [3]. However, availability, costs, and lack of sufficient standardization and evaluation of the simulation resulted in the prolongation of the learning curve of the surgeons. In laparoscopic surgery, virtual reality simulation training has been established as an effective method for improving laparoscopic surgical performance among trainees [4,5]. 

As the objective of the present study, we evaluate the benefit of training with virtual reality simulations, determining whether training improves the skill acquisition of the candidates independent of the previous level of experience. The secondary objective was to describe the short-term skill acquisition obtained by simulation training and to determine the factors affecting its magnitude.

## 2. Materials and Methods

### 2.1. Subjects

Between June 2017 and July 2018, we prospectively enrolled consecutive participants who agreed to participate in the study among trainees who received laparoscopic training at the ALEXEA (Alexandria Endoscopy Association) Center, in collaboration with the Department of Gynecology and Obstetrics, University Hospitals, Campus Kiel Schleswig-Holstein, Germany. The Institutional Review Board of ALEXEA granted ethics approval for the study and all participants signed an informed consent. Before starting their training sessions, participants filled out a questionnaire addressing their demographic data, including age, sex, current occupation, and experiences in laparoscopic surgery as a camera assistant (CA EXP), first assistant (FA EXP), and surgeon (S EXP). 

### 2.2. Evaluation Process

With no consensus for an evaluation procedure having been established, we decided to follow the evaluation process suggested by previous publications evaluating virtual reality simulators [6,7]. This process is divided into three steps: the first is face evaluation, which demonstrates the acceptance of the design and realism of the tasks (Appendix A). The second is constructive evaluation, which demonstrates the improvement of the participants in the training session and the ability of the designed task to distinguish the experienced from the inexperienced surgeon based on the performance score. The third step is predictive evaluation, which demonstrates the beneficiary effect of training four weeks afterward on the practice of surgeons addressed with five questions [8,9] (Appendix A).

In order to mimic the actual use of the simulator in a training curriculum and to establish baseline theoretical knowledge, participants were required to watch a self-guided teaching tutorial before starting the task. The participants performed two different tasks twice. Each participant was allowed to practice alone twice before performing a post-test, as the first trial is used to get accustomed to the simulator. The training curriculum for the second session was identical to the first session. The score and time needed to achieve the desired task were automatically documented. 

Immediately after finishing the training session, the participants filled out a detailed questionnaire based on an evaluation scale ranging from 1 (least) to 10 (most), addressing the face evaluation of the simulator and the desired task. The face evaluation was addressed by five questions regarding participants’ personal impressions of the simulator and the design of the simulator. The feedback on the realism of the task was addressed by addressing the participants’ personal assessment of the task, coordination of the instruments, and tissue feedback. 

Four weeks after finishing the training, the participants filled out the questionnaire addressing the predicative evaluation of the simulator. The beneficial effect of training on the real practice of surgeons was addressed with five questions regarding the implementation into their daily practice, performance of laparoscopic procedure, laparoscopic suturing, shortening of the operative time, and management of complications. 

To evaluate constructive evaluation, we observed whether the initial time and score could distinguish experienced surgeons from inexperienced ones. Participants’ experience was assessed objectively and subjectively. The objective assessment includes the professional status and years of experience. The subjective assessment was obtained by asking every surgeon to evaluate his or her own experience as a camera assistant (CA EXP), first assistant (FA EXP), and surgeon (S EXP), and his/her experience with video games (video game EXP). The participants evaluated themselves on a score from 1 to 10, 1 being completely inexperienced and 10 being highly experienced. 

Change in time and score in tasks I and II were used as a measure for short-term skill acquisition. They were calculated by subtracting the time and score achieved in the second session from those of the first session. Factors that could affect short-term skill acquisition include the age of the participant, initial time, initial score, and different measures of experience.

### 2.3. Apparatus

The simulator setup was a VR laparoscopy simulator (LapSim) with haptic feedback, Surgical Science AB, Gothenburg, Sweden. 

**Figure 1:**
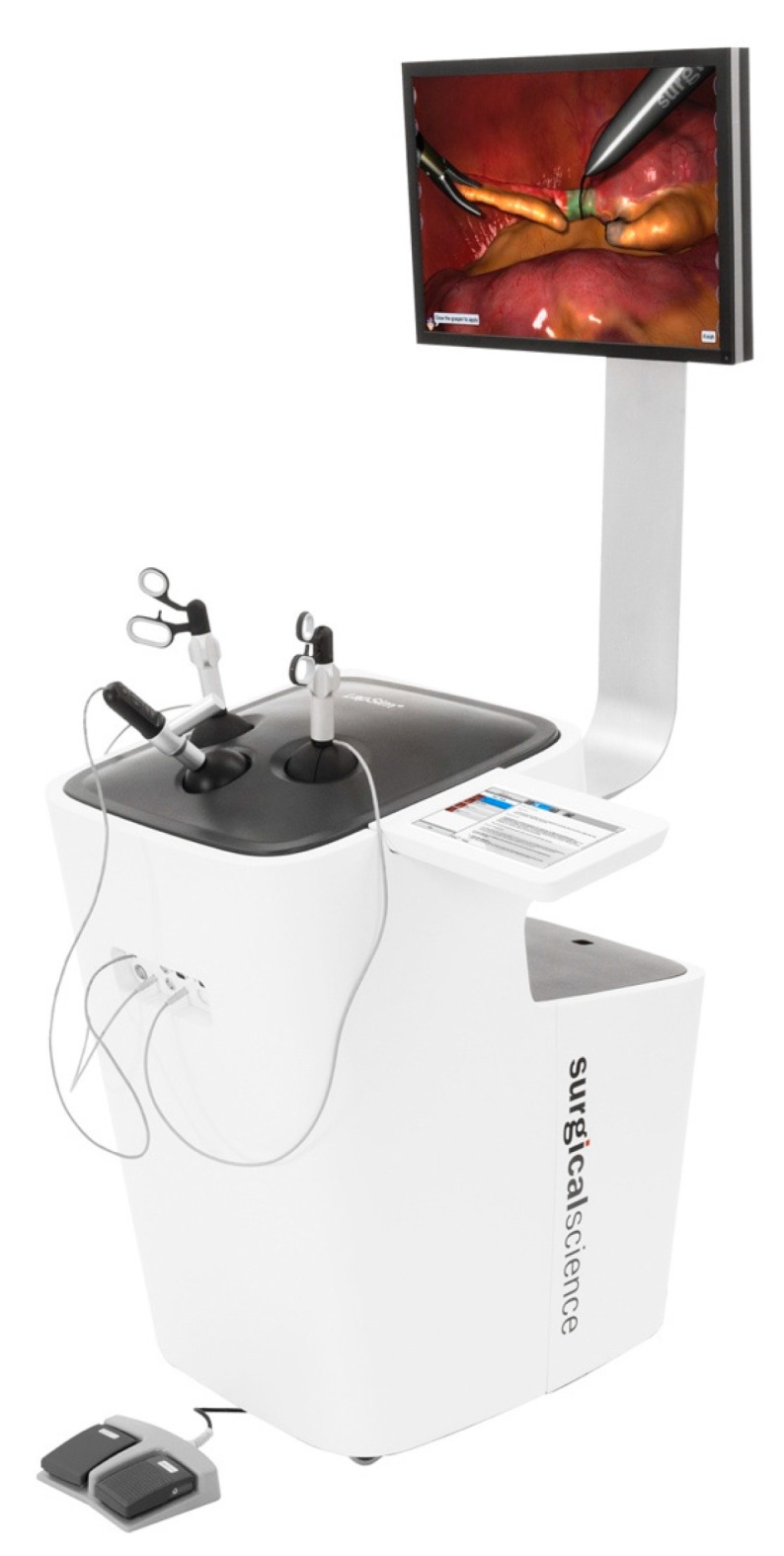
Shows the virtual reality simulator.

LapSim consisted of a 27-Inch LCD monitor, a keyboard and mouse, a windows PC, and Simball ^TM^ 4D joysticks, with a double footswitch. For the interaction with the VR environment, the simulator provided Sim-ball 4D joysticks, which had laser-marked ball joints, with three degrees of freedom that allowed real-time calculations of the exact 3D angular position. The input devices included a grasping instrument on the left and right sides and a camera instrument in the center (Figure 1).

### 2.4. Tasks

The participants performed two tasks. Task I was to transfer the pegs with both instruments and task II was a salpingectomy by an extra-uterine pregnancy. Each participant performed the chosen task twice and two parameters were documented: total time (seconds) and score (% age).

### 2.5. Multimetric Score System

The overall results were shown as the sum of the assessment of execution quality for each performed task. The assessment was automatically calculated using the LapSim software. The scoring system recorded points for the successful accomplishment of the assigned task and simultaneously subtracted points for errors. 

### 2.6. Statistical Analysis

The data were collected in a database and analyzed using all trainee data sets. The performance parameters were recorded using the simulator software and output files were created using Microsoft Excel (Microsoft Corp, Redmond, WA, USA). SPSS version 25 (IBM, Armonk, NY, USA) was used to log and analyze the data. Median and interquartile range were used for statistical analysis. A Wilcoxon-signed rank test was used to compare time and score between the pre-test and the post-test. Spearman’s rho test was used to test correlations among quantitative and qualitative ordinal variables. Cronbach’s alpha assessed the consistency of the questionnaires developed to evaluate face evaluation and predictive evaluation. We received statistical support from the Department of Statistical Analysis, University of Alexandria.

## 3. Results

### 3.1. Baseline Characteristics

The participants had a median age of 35 years and 94% of them (*n* = 59) were right-handed. The study population was represented with three different groups of experience: 16% (*n* = 10) were residents, 46% (*n* = 29) were specialists, and 38% (*n* = 24) were consultants with median experience of three years as a consultant (expert group) in laparoscopy (Table 1).

### 3.2. Face Evaluation

The participants had high overall impressions of the LapSim, showing a median score of eight (IQR 3), 57% (*n* = 36 participants) highly appreciated the Lap-Sim, 38% (*n* = 24) moderately appreciated it, and only 5% (*n* = 3) showed low satisfaction. 

The design of the Lap-Sim was highly evaluated, showing a median score of eight (IQR 3); 67% (*n* = 42 participants) were highly satisfied with the design of the task and 32% (*n* = 20) showed a moderate satisfaction with the design (Table 2). 

The evaluations of the different aspects of realism were addressed through the questionnaire concerning the tissue feedback, instrument manipulation, and assessment of the tasks; the realism of those aspects had median ratings of seven (IQR 3), eight (IQR 4), and seven (IQR 4), respectively. Forty-nine percent (*n* = 31) and 54% (*n* = 34) of participants rated the assessment of the task and the instrument manipulation highly, but the majority of the participants, 54%, (*n* = 34) rated the tissue feedback with a moderate score (Table 2). 

The questionnaire addressing the face evaluation showed good consistency and statistically measured a good variance shown by the Cronbach’s alpha of 0.69 (Table 2).

The participants had high overall impressions of the LapSim, showing a median score of eight (IQR 3), 57% (*n* = 36 participants) highly appreciated the Lap-Sim, 38% (*n* = 24) moderately appreciated it, and only 5% (*n* = 3) showed low satisfaction.

### 3.3. Constructive Evaluation and Short-Term Skill Acquisition

The participants accomplished task I at a median of 187 s (IQR 66.6) in the pre-test and 149 s (IQR 61.4) in the post-test. Improvements were recorded by 75.4% (*n* = 46) participants, showing a significant 20% (42.1 s) reduction between the two trials. The pair-wise comparison (*Z*) and the correlation (*p*) showed a significant statistical significance (*p* < 0.001) (Table 3 and Table 4).

The median score (IQR) at task I in the pre-test was 50 (27.0) and 72 (28.5) in the post-test. Improvements were recorded by 90.2% (*n* = 55) of participants, showing a significant improvement in the score of 15% (nine points) between the two trials. The pair-wise comparison (*Z*) and the correlation (*p*) showed a significant statistical significance (*p* < 0.001) (Table 4).

At task II (laparoscopic salpingectomy), the participants accomplished the desired task in the pre-test at a median of 477 s (IQR 229.4) and 410 s (IQR 123.5) in the post-test. Improvements were recorded by 70.5% (*n* = 43) of participants, showing a significant reduction of 15% (33 s) between the two trials. The pair-wise comparison (*Z*) and the correlation (*p*) showed a significant statistical significance (*p* < 0.001) (Table 4). 

Moreover, the median score (IQR) for task II in the pre-test was 56 (16.5), which improved to 66 (20.0) in the post-test. A high proportion of participants (78.7%, *n* = 48) improved, recording a significant improvement in score by 8.1% (24 points) between the two trials. The study showed that those with high scores in the pre-test were able to score high in the post-test, showing a significant pair-wise comparison (*Z*), and the correlation (*p*) showed significant statistical significance (*p* < 0.001) (Table 3).

The median score (IQR) at task I in the pre-test was 50 (27.0) and 72 (28.5) in the post-test. Improvements were recorded by 90.2% (*n* = 55) of participants, showing a significant improvement in the score of 15% (nine points) between the two trials. 

At task II (laparoscopic salpingectomy), the median score (IQR) for task II in the pre-test was 56 (16.5), which improved to 66 (20.0) in the post-test. A high proportion of participants (78.7%, *n* = 48) improved, recording a significant improvement in the score of 8.1% (24 points) between the two trials.

The scores of tasks I and II showed a significant positive correlation with surgeons’ experiences as camera assistants (*p* = 0.289, *p* = 0.024, and *p* = 0.306, *p* = 0.017, respectively). Participants who perceived themselves as expert camera assistants had higher scores in tasks I and II. At task I and II (laparoscopic salpingectomy), the pair-wise comparison (*Z*) and the correlation (*p*) showed a significant statistical significance (*p* < 0.001).

The scores of tasks I and II showed a significant positive correlation with surgeons’ experiences as camera assistants (*p* = 0.289, *p* = 0.024, and *p* = 0.306, *p* = 0.017, respectively). Participants who perceived themselves as expert camera assistants had higher scores in tasks I and II. The accomplished time for task I showed a significant negative correlation with participants’ experience as first assistants and in video games (*p* = −0.281, *p* = 0.028, and *ρ* = −0.470, *p* < 0.001, respectively). 

**Figure 2:**
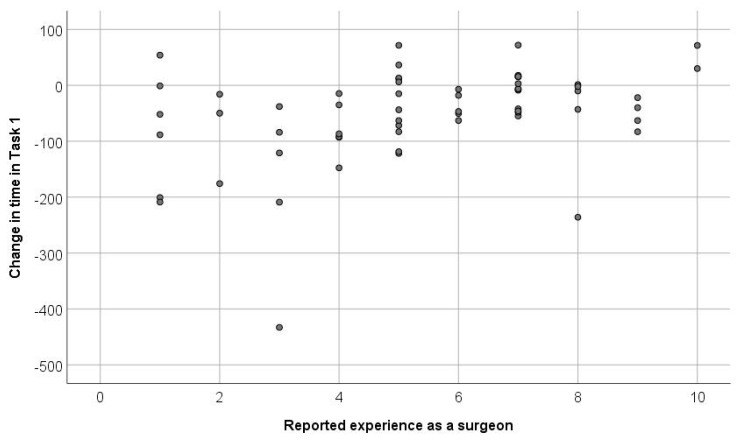
Shows the factors affecting the magnitude of short-term skill acquisition; participants who had more experience as camera assistants, surgeons, and in video games achieved more gain in task I. Also, a shorter initial time and higher initial score predicted higher gains in both tasks.

**Figure 3:**
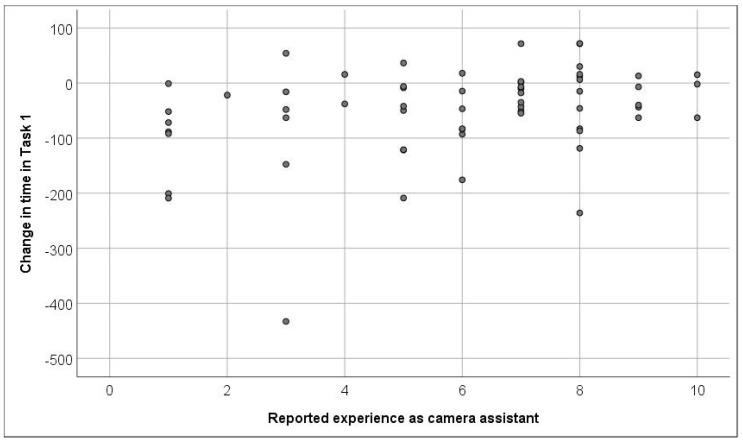
Shows the factors affecting the magnitude of short-term skill acquisition, participants with fewer years of experience as camera assistants excelled in reducing the time taken to complete the task given, as shown by the directly proportional relationship between the initial time and the value of time reduced between trials.

The recorded times in the tasks were significantly shorter in the second session than in the first one (*p* < 0.001). Also, the scores of the two tasks were significantly higher in the second session. Figure 2 and Figure 3 show the factors affecting the magnitude of short-term skill acquisition: participants who had more experience as camera assistants, surgeons, and in video games achieved more gain in task I. Also, a shorter initial time and higher initial score predicted higher gains in both tasks.

Participants with fewer years of experience as a surgeon excelled in reducing the time taken to complete the task given, as shown by the directly proportional relationship between the initial time and the value of time reduced between trials.

### 3.4. Predicative Evaluation 

Four weeks after finishing the training with the simulator, the beneficial effect of training on the real practice of surgeons was addressed with five questions. The surgeons’ rating showed an improvement regarding the implementation into their daily routine, performance of laparoscopic procedure, laparoscopic suturing, shortening of the operative time, and management of complication, with median scores of seven (IQR 9), seven (IQR 9), six (IQR 9), six (IQR 8), and five (IQR 8), respectively (Table 5).

Forty-two percent (*n* = 25) and 49% (*n* = 29) of participants gave high ratings regarding their improvement in terms of implementation into their daily routine and performance of laparoscopic procedures. However, the majority noted low benefits regarding the management of complications (46%, *n* = 27) and shortening of operative time (44%, *n* = 26). 

The questionnaire addressing the predicative evaluation showed a strong consistency and statistically measured a strong variance shown by the Cronbach’s alpha value (0.095) (Table 5).

## 4. Discussion

The study design selected two tasks evaluating different training dimensions. Task I mainly addressed basic coordination skills and tactile feedback from the simulator, which is the milestone for laparoscopy. Task II (laparoscopic salpingectomy for extra-uterine pregnancy) addressed several training integrals: anatomy, patient safety, and a real situation dealing with operative stress and complications, bleeding, and fatigue by prolonged operative time. 

The time devoted to the teaching of surgery is being reduced more and more, necessitating the development of teaching practices outside of the operating room [10,11], which shows the need for a good simulation model, resembling a high similarity to the laparoscopic procedures. A recent metanalysis published by Portelli, showed a statistical advantage at the OSATS for the surgical trainees using the virtual reality; the OSATS score which is a total of seven parameters including respect for tissue, time and motion, instrument handling, knowledge of instruments, flow of operation, formed planning, and knowledge of the specific procedure. Four RCTs were chosen consisting of a total of 99 participants (50 in the virtual reality training group vs 49 in the control group [2].

In the present study, face evaluation for the LapSim is established with outstanding ratings for acceptance of the design and realism of the tasks. We are aware that the rating for the face evaluation is subjective and influenced by several factors, such as an enthusiastic presentation of the simulator and the motivation of participants to improve performance, consistency of the performance, and the decrease in the number of errors. Nevertheless, the rating for training capacity and realism presented in other studies for different simulator systems are varying in the convincing [11,12], recently several 3D portable simulators, although not being able to demonstrate differences between the performance scores between experts and surgical novices, are still showing good consistency and reliability [9]. 

Although the literature considers haptic feedback as a controversial topic in surgical simulation [7,13], the LapSim received positive evaluations regarding tissue feedback, instrument manipulation, and assessment of the tasks, which can also reflect a potential for a good training capacity. 

Based on the work of Grantcharov [14] and Gallagher and Satava [8], it is suggested that more repetitions may better reflect a subject’s true baseline performance, rather than relying on a single performance. The predefined repetitions were designed carefully to allow, through the pre-test, a suitable warm-up exercise and to acquaint the participants with the simulator itself, and the post-test to access the learning curve and effect, also avoiding over-repetition to avoid the bias of familiarization with the simulator itself. 

The majority of the participants were able to show an improvement in time and score for both tasks, showing acceptable learning curves for both tasks, reflecting a good constructive evaluation of the training. 

From our results, it seems that participants with fewer years of experience excelled in reducing the time taken to complete the tasks given (Figure 2 and Figure 3), which is shown with the directly proportional relationship between the initial time and the value of time reduced between trials. This could be explained by the fact that less experienced operators had a stronger desire and motivation to train and improve. This agreed with studies by the University of Michigan [15] and the University of Kiel [5,6] with similar simulators, which showed less experienced groups having an initial advantage and greater margin of improvement. Moreover, the ability to train independently with the simulator, setting the desired operative scenarios and difficulty, and receiving an automated detailed feedback on the performance allows the participants a certain degree to flexibly set the training time goals of every session. 

Uniquely, our study addresses the predicative evaluation of the simulator; the transfer of skills and concurrent improvement in the operative performance of the participants for the first four weeks after finishing the training. The participants’ feedback shows that the simulator training enabled them to improve the implementation in their daily routine and performance of laparoscopic procedures. The study also showed a positive relationship between being satisfied with the design of the LapSim and scoring high in terms of improvements in their performance at the operative room. This agrees with several studies of laparoscopic and hysteroscopic simulation training, which show improvement in the operative room for a period of one month after training, but a decreasing effect after six months [14,15]. Mathews et al. emphasized the value of validated laparoscopic virtual simulation tasks and correlated it with surgical volume and characteristics of practicing gynecologists as a predictor for objective performance [1]. 

The simulator allows the participants the chance to train individually with the different operative scenarios adjusting the difficulty level without the interference of an instructor the whole time during the training session, permitting the participant to receive detailed feedback of the performance based on an automated scoring system. 

Adding sources of disturbances while training would have been a good addition to our training environment, which is considered to be one of the limitations of our study, however recent studies showing that the impact of acoustic disturbance on the performance of surgeons is noted by experienced surgeons [10]. Recent publications and innovations from the University of Mainz in Germany, combining the VR (virtual reality) with head-mounted displays (HMDs) allowing the participant to be trained for different sources of disturbance, which shows the need for future technical research to improve the visualization and capability of interacting with a virtual scenario [16]. 

## 5. Conclusions

Based on the three stages of evaluation, the virtual simulation with LapSim can help in teaching basic skills in the early stages of training and provide a good simulation for procedural operation for resident and fellowship training. The virtual simulation demonstrated significant results in most parameters; reducing operating time, improvement of tissue handling, instrument coordination, and reducing the incidence of complications resulting in an improvement in patient safety. 

Future studies are still needed in order to incorporate more realism and virtual scenarios dealing with complications into the standard surgical training curriculum.

## Figures and Tables

**Table 1:** Demographic characteristics of the study participants.

Participant Characteristic	Statistical Summary *	
Age	36	(10.0)
Male, *n* (%)	55	(87)
Rt hand, *n* (%)	59	(94)
Measures of experience		
Professional Status, *n* (%)		
Resident	10	(16)
Specialist	29	(46)
Consultant	24	(38)
Years of experience	3	(7.0)
Perceived experience as		
Camera assistant	6	(4.0)
First assistant	7	(3.0)
Surgeon	5	(3.0)
In video games	4	(6.0)
Initial performance		
T.1 E1 time	188	(68.2)
T.1 E1 score	55	(28.0)
Two trials, *n* (%)	61	(97)
T.2 E1 time in minutes	149	(61.4)
T.2 E1 score	72	(28.5)

* All values are median and interquartile range (IQR) unless otherwise is specified.

**Table 2:** Face evaluation of simulation.

Item	Median (IQR)	High (8:10)	Moderate (5:7)	Low (<5)
		*n*	*n*	*n*
Face Evaluation				
Tissue feedback	7	23	34	6
	(3)	(37%)	(54%)	(10%)
Assessment of the procedure	7	31	30	2
	(4)	(49%)	(48%)	(3%)
Impressions of the LapSim	8	36	24	3
	(3)	(57%)	(38%)	(5%)
Design of LapSim	8	42	20	1
	(3)	(67%)	(32%)	(2%)
Instrument manipulation	8	34	25	4
	(4)	(54%)	(40%)	(6%)
Cronbach’s = 0.69				

**Table 3:** Constructive evaluation of LapSim simulator.

Parameter	Task I (*n* = 61)		Task II (*n* = 61)	
Time in minutes				
First session	187.0	(66.6)	477.0	(229.4)
Second session	149.4	(61.4)	410.0	(123.5)
Pair-wise comparison, *Z (p*-value)	4.6	(<0.001)	4.4	(<0.001)
Correlation, *p* (*p*-value)	486	(<0.001)	474	(<0.001)
Change *				
Absolute	−42.1	(83.7)	−71.7	(187.5)
Percentage from the 1st duration	−20.2	(36.9)	−15.3	(33.2)
Reduction in time, *n* (%)	46	(75.4)	43	(70.5)
Score				
First session	50.0	(27.0)	56.0	(16.5)
Second session	72.0	(28.5)	66.0	(20.0)
Pair-wise comparison, *Z* (*p*-value)	5.1	(<0.001)	4.2	(<0.001)
Correlation, *p* (*p*-value)	711	(<0.001)	655	(<0.001)
Change *				
Absolute	9.0	(14.5)	5.0	(14.0)
Percentage from the 1st score	15.3	(33.4)	8.1	(24.0)
Increase in score, *n* (%)	55	(90.2)	48	(78.7)

Values are median and (IQR) unless otherwise specified. * Change is calculated by subtracting the observation of the second session from that of the first session.

**Table 4:** Improvement in performance in terms of time and score between the two sessions.

Factor	Time (*n* = 61)		Score (*n* = 61)	
		(*p*-Value)		(*p*-Value)
Task I				
Professional status	0.128	(0.327)	−0.284	(0.027)
Year of experience	−0.125	(0.337)	0.038	(0.770)
Perceived experience as				
Camera assistant	0.038	(0.773)	0.289	(0.024)
First assistant	−0.281	(0.028)	0.163	(0.211)
Surgeon	−0.144	(0.268)	−0.123	(0.345)
In video games	−0.470	(<0.001)	−0.068	(0.601)
Task II				
Professional status	0.069	(0.598)	−0.194	(0.133)
Year of experience	−0.059	(0.651)	0.065	(0.620)
Perceived experience as				
Camera assistant	0.014	(0.913)	0.306	(0.017)
First assistant	−0.113	(0.386)	0.216	(0.094)
Surgeon	−0.150	(0.249)	0.007	(0.958)
In video games	−0.221	(0.087)	−0.006	(0.963)

**Table 5:** Predictive evaluation of simulation.

Predictive Evaluation				
Daily routine	7	25	12	22
	(9)	(42%)	(20%)	(37%)
Performance of laparoscopic procedures	7	29	8	22
	(9)	(49%)	(14%)	(37%)
Laparoscopic suturing	6	23	13	23
	(9)	(39%)	(22%)	(39%)
Shortening of the time of operation	6	16	17	26
	(8)	(27%)	(29%)	(44%)
Management of complications	5	23	9	27
	(8)	(39%)	(15%)	(46%)
Cronbach’s = 0.95				

## Data Availability

Data sharing not applicable. No new data were created or analyzed in this study. Data sharing is not applicable to this article.

## Appendix A

The documentation sheet for documenting the face evaluation, which demonstrates the acceptance of the design and realism of the tasks is provided. The second documentation sheet is the predictive evaluation sheet collected after performing the desired tasks, which demonstrates the beneficial effect of training four weeks after the practice of surgeons addressed with five questions.
